# Integration-Associated Paraspinal Neoplasm Following AAV Gene Therapy

**DOI:** 10.1056/NEJMc2607927

**Published:** 2026-10-15

**Authors:** Josh N. Vo, Yi-Mi Wu, Amer Heider, Xuhong Cao, Rui Wang, Tiffany Pham, Guo Ci Teo, Suresh N. Magge, Hemant Parmar, Rajen Mody, Dan R. Robinson, Arul M. Chinnaiyan

**Affiliations:** 1 Michigan Center for Translational Pathology, Ann Arbor, MI; 2 University of Michigan Medical School, Ann Arbor, MI; 3 C.S. Mott Children’s Hospital, Ann Arbor, MI

## TO THE EDITOR:

A 3-year-old boy with infantile-onset Krabbe disease (biallelic *GALC* variants p.Arg220Ter and p.Tyr319Cys) underwent two sequential umbilical-cord-blood transplantations (UCBTs), at 5 and 9 months, each after myeloablative, alkylator-based conditioning. On day +29 after the second UCBT he received intravenous FBX-101 (RESKUE trial, NCT04693598),^1^ an AAVrh.10-capsid vector with AAV2 inverted terminal repeats carrying *GALC* under a CMV-enhancer/chicken β-actin (CAG) promoter, at 3×10^13^ vector genomes per kilogram. He remained poorly immune-reconstituted, requiring donor lymphocyte infusion and immunosuppression for graft-versus-host disease (Figure 1A).

Twenty-six months later, surveillance MRI revealed an incidental L2-L3 paraspinal mass. Biopsy showed a small round-blue-cell neoplasm with focal epithelioid, nested morphology, positive for CD56 and S100 and strongly positive for GLI1 by in situ hybridization, with a Ki-67 index below 10%; the diagnosis was a GLI1-altered neoplasm.^2^ *GLI1* mRNA ranked in the 100th percentile of our 5,143-sample cohort (Figure 1B), with concordant *PTCH1* overexpression, and targeted sequencing showed a stable diploid genome without oncogenic mutations or fusions. However, Oxford Nanopore long-read sequencing identified a clonal 1,038-bp insertion (44% of reads) in *GLI1* intron 1, flanked by 115-bp target-site duplications (Figure 1C). The integrant was a subgenomic vector fragment comprising a partial AAV2 ITR, the CMV enhancer, the chicken β-actin promoter, exon 1, and a splice donor, lacking the *GALC* transgene and polyadenylation signal. The splice donor generated a chimeric transcript joining chicken β-actin exon 1 to *GLI1*, driving full-length *GLI1* expression, and allele-specific hypomethylation confined to the integrant allele established the integration as the proximate cause of *GLI1* activation (Supplementary Appendix).

AAV vectors are predominantly nonintegrating and have an established safety record across thousands of recipients;^3^ nonetheless, we report this case of AAV-integration-driven oncogenesis shortly after the publication of report of *PLAG1*-activated tumor by Ahrens-Nicklas et al..^4^ Both integrants were truncated, rearranged subgenomic structures that retained a CAG promoter-enhancer and splice donor and activated a developmental transcription factor. Unlike the MPS I integrant, which retained fragments of the *IDUA* transgene, the integrant we describe here had no transgene or polyadenylation sequence, raising concern that a “snapback” or promoter-only species arising during vector manufacturing was the integrating substrate.^5^ A potential contribution of this patient’s transplant setting, which involved a genotoxic regimen and immunosuppression, is discussed in the Supplementary Appendix.

The event was promptly reported to the treating physicians and the FDA. The patient remains asymptomatic. These findings argue for splice-donor-free cassette design, attention to vector purity, and extended genomic surveillance after AAV gene therapy.

## Supplementary Material

Supplementary Appendix

## Figures and Tables

**Figure 1.**
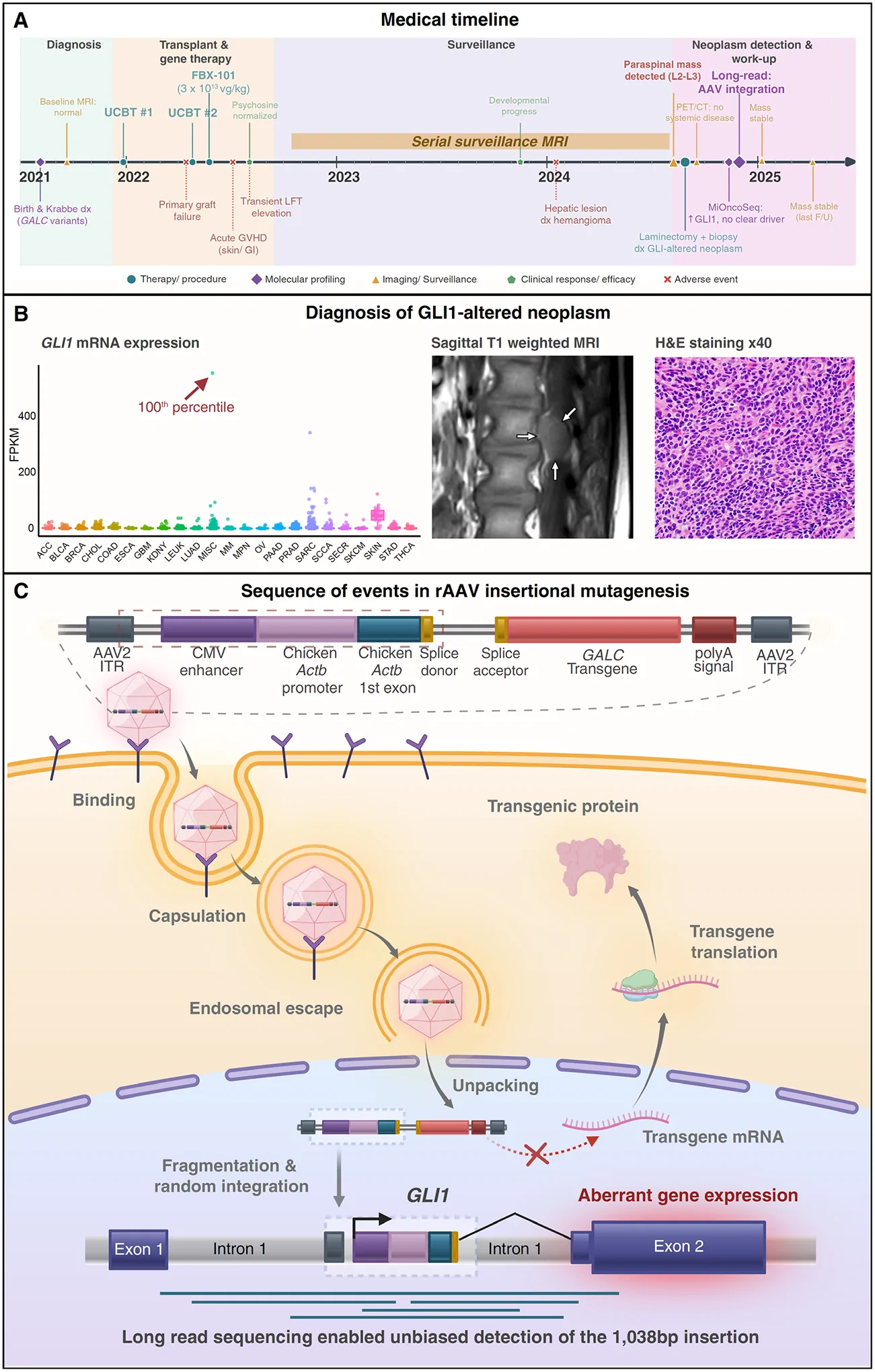
(**A**) Clinical timeline from Krabbe diagnosis through two umbilical-cord-blood transplantations, FBX-101 gene therapy, and detection of the paraspinal neoplasm. (**B**) Tumor *GLI1* expression (red arrow) versus a pan-cancer cohort, sagittal T1-weighted MRI of the L2-L3 mass (arrows), and hematoxylin and eosin staining (×40). (**C**) A subgenomic vector fragment (CMV enhancer, chicken β-actin promoter, first exon, and splice donor; lacking the *GALC* transgene and polyadenylation signal) integrated into *GLI1* intron 1 and spliced to exon 2, driving aberrant *GLI1* expression, detected as a 1,038-bp insertion by long-read sequencing.

## References

[1] Escolar M, Lugt MV, Poe M, Greco M, Werling K, De Silva E, P014: First-in-human phase 1/2 trial of intravenous FBX-101 with cord blood transplantation increases GALC, brain and motor development in infantile Krabbe. Genet Med Open. 2023;1(1):100024.

[2] Antonescu CR, Agaram NP, Sung YS, Zhang L, Swanson D, Dickson BC. A Distinct Malignant Epithelioid Neoplasm With GLI1 Gene Rearrangements, Frequent S100 Protein Expression, and Metastatic Potential: Expanding the Spectrum of Pathologic Entities With ACTB/MALAT1/PTCH1-GLI1 Fusions. Am J Surg Pathol. 2018;42(4):553–560. doi:10.1097/PAS.0000000000001010 PMC5844813 29309307

[3] Byrne BJ, Flanigan KM, Matesanz SE, Finkel RS, Waldrop MA, D’Ambrosio ES, Current clinical applications of AAV-mediated gene therapy. Mol Ther. 2025 June 4;33(6):2479–516. 10.1016/j.ymthe.2025.04.045 PMC12172329 40329530

[4] Ahrens-Nicklas RC, Kotch C, Roche AM, Everett JK, Reddy S, Santi M, Neuroepithelial tumor with AAV integration after intracisternal magna vector delivery. N Engl J Med. 2026 May 13. doi:10.1056/NEJMoa2601608 42127296

[5] Brimble MA, Winston SM, Davidoff AM. Stowaways in the cargo: contaminating nucleic acids in rAAV preparations for gene therapy. Mol Ther. 2023;31(10):2826–2838. 10.1016/j.ymthe.2023.07.025 PMC10556190 37533254

