# Supplementary material for: Integration-Associated Paraspinal Neoplasm Following AAV Gene Therapy
Source: N Engl J Med. Author manuscript; Available in PMC 2026 Oct 15. (PMC13671263; doi:10.1056/NEJMc2607927)
Supplement: Supplementary file 1 [file NIHMS2203103-supplement-Supplementary_Appendix.pdf]

Table of Contents

Background 2

Case Presentation 3

Supplementary Discussion 9

Supplementary Methods 15

- **Regulatory Oversight** 15
- **Integrative clinical sequencing by the Illumina platform** 15
- **Long-read sequencing protocol** 16
- **RiboErase RNA sequencing** 16
- **Short-read and long-read sequencing analyses** 17

Supplementary Figures 19

- **Figure S1: Additional MRI results of the spinal mass.** 19
- **Figure S2: Tumor Histopathology and Immunohistochemistry** 20
- **Figure S3: Short-read copy number analysis.** 21
- **Figure S4: Haplotype phasing for *GALC* variants.** 22
- **Figure S5: Genomic characterization of AAV vector integration.** 23
- **Figure S6: Orthogonal validation of rAAV insertion by PCR** 24
- **Figure S7: RNA sequencing analysis confirms aberrant GLI1 splicing** 25
- **Figure S8: Allele-specific methylation analysis of the AAV integration site.** 26

Supplementary References..27

Background

Krabbe disease, also known as globoid cell leukodystrophy, is a rare autosomal recessive disorder caused by mutations in the galactosylceramidase (*GALC*) gene. GALC deficiency leads to the accumulation of galactosylsphingosine (psychosine), causing widespread demyelination and neurodegeneration in both the central and peripheral nervous systems.[1,2] The most severe form is early infantile Krabbe disease, manifesting before 6 months of age with symptoms including irritability, spasticity, developmental regression, and seizures.[1,2] Without treatment, the disease rapidly progresses to death, typically by 2 years of age. Hematopoietic stem cell transplantation (HSCT) is the standard of care for patients with infantile Krabbe disease and preserves cognitive function; however, peripheral nerve disease continues to progress over time, leading to variable degrees of motor dysfunction.[3,4] In addition, its effectiveness is largely limited to pre-symptomatic patients, the majority of whom are now identified through newborn screening.[5,6]

Adeno-associated virus (AAV)-mediated gene therapy has emerged as a promising treatment strategy for Krabbe disease.[7] AAV vectors can effectively cross the blood-brain barrier and provide stable gene expression in the central nervous system (CNS), making them particularly suitable for treating neurological disorders.[8] Preclinical studies using AAV vectors carrying the *GALC* transgene have demonstrated significant improvements in survival and motor function in animal models.[9] Most notably, a recent study using a canine model demonstrated that combining HSCT with AAV gene therapy produced superior outcomes compared to either treatment alone.[10]

The study revealed dose-dependent improvements in neurological function, confirming both safety and efficacy of this approach in an animal model and forming the basis for two clinical trials in humans.[10]

The safety profile of AAV-based therapy requires careful consideration. One concern is the immune response against the AAV vector or the therapeutic protein it delivers.[11] Such immune reactions can lead to inflammation, tissue damage, or the destruction of transduced cells, thereby compromising the efficacy of the therapy.[11] Additionally, the long-term effects of AAV integration into the host genome are not fully understood, raising questions about potential insertional mutagenesis and oncogenesis.[12] While AAV is considered safer than other viral vectors in terms of oncogenic potential due to the vector remaining primarily episomal, AAV vectors have been shown to be able to integrate at sites of existing chromosomal breaks *in vitro.*[13] Studies in mice have also highlighted risks of hepatocellular carcinoma (HCC) following AAV gene therapy.[14] However, until very recently, no definitive case of tumor caused by AAV integration had been reported in humans; one such case has now been described in a patient with mucopolysaccharidosis type I.[15] For this reason, current FDA guidance for follow-up after gene therapy recommends up to 5 years of follow-up for AAV vectors, compared with 15 years for other forms of gene therapy.[16] Here, we report a case of a patient developing a tumor following AAV therapy in a clinical trial—a 3-year-old boy who received treatment for Krabbe disease.

Case Presentation

A 3-year-old male diagnosed with infantile-onset Krabbe disease was identified through state-wide newborn screening. Genetic testing revealed compound heterozygous pathogenic *GALC* variants,

c.658C>T (p.Arg220Ter) and c.956A>G (p.Tyr319Cys), along with homozygosity for the pseudodeficiency variant c.742G>A (p.Asp248Asn).[2] At diagnosis, his galactocerebrosidase activity was markedly reduced at 0.23 μmol/L/hr, and his psychosine concentration was elevated at 2 nM on dried blood spot analysis. Initial magnetic resonance imaging (MRI) at 2 weeks of age was unremarkable, and follow-up at 2 months showed no change. Audiologic evaluation at 2 weeks of age was normal; however, repeat testing at 2 months revealed abnormal outer/middle ear function on tympanometry and absent emissions on distortion product otoacoustic emissions (DPOAE) in the left ear, while the right ear remained normal. Auditory brainstem response testing was abnormal bilaterally, with greater impairment on the left side. On clinical examination, the patient exhibited bilateral shoulder girdle weakness, hypotonia, and head lag, suggesting infantile-onset disease (Figure 1A).

The patient underwent umbilical cord blood transplantation (UCBT) using a 5/6 HLA-matched cord blood unit at 5 months of age following a myeloablative conditioning regimen of hydroxyurea, alemtuzumab, fludarabine, and busulfan. This was complicated by veno-occlusive disease of the liver, infection with SARS-CoV-2, and poor graft function despite donor lymphocyte infusion (DLI) on day +42 post-UCBT. Unfortunately, he ultimately met criteria for primary graft failure (Figure 1A). He then underwent a successful second 5/6 HLA-matched UCBT at 9 months of age (4 months after the first transplant) following conditioning with hydroxyurea, alemtuzumab, fludarabine, melphalan, and thiotepa. This transplant was complicated by hyperacute graft-versus-host disease (GVHD) and engraftment syndrome at 11 days post-UCBT (Figure 1A).

The patient was then enrolled in a phase 1/2 study evaluating the safety and efficacy of an adeno-associated virus (AAV) gene replacement therapy for Krabbe disease administered in combination

with UCBT.[17] He received 3×10^{13} vector genomes per kilogram of body weight of gene therapy vector intravenously on day +29 post-UCBT. The vector was an AAVrh.10-capsid vector with AAV2 inverted terminal repeats that carries GALC under a CAG promoter (a cytomegalovirus [CMV] enhancer, chicken β-actin [Actb] promoter, and splice donor). A DLI was administered on day +55 post-UCBT (day +26 post gene therapy (GT)) due to poor immune reconstitution. He subsequently developed acute cutaneous and gastrointestinal GVHD on day +70 post-UCBT (day +41 post-GT) requiring treatment with methylprednisolone and ruxolitinib. The patient also experienced transient liver enzyme elevation (peak AST 292 U/L, ALT 389 U/L, and GGT 1,817 U/L), which resolved after switching from voriconazole to fluconazole, with no coagulopathy or jaundice. In addition, at 18 months post-GT, surveillance imaging identified a 1.8 cm hepatic lesion in segment 5. Alpha-fetoprotein (AFP) was transiently elevated, raising concern for hepatocellular carcinoma; however, liver biopsy confirmed a hemangioma. The lesion has remained stable on subsequent imaging.

Routine surveillance MRI performed at 26 months post-GT identified an incidental finding of a new 1.5 x 0.9 x 1.5 cm paraspinal mass at the L2-L3 level, extending from the epidural space into the left neural foramen (Figure 1B and Figure S1). A computed tomography (CT) scan of the chest, abdomen, and pelvis revealed no other masses. A fine needle aspiration revealed a small blue cell neoplasm with high nuclear-to-cytoplasmic ratio and monotonous morphology, but an initial immunohistochemical workup failed to provide a definitive diagnosis.

Neurosurgery was thus consulted, and an open biopsy was scheduled, with the patient undergoing an L2 laminectomy with an intradural tumor biopsy. The lesion appeared fibrous and localized to the left L2 nerve root and was generously sampled but not fully resected to preserve neurological

function. The histological sections revealed a relatively monotonous small round blue cell neoplasm with focal epithelioid morphology infiltrating ganglion and soft tissue [H&E (hematoxylin and eosin stain), 10x] (Figure 1B and Figure S2). The Ki-67 proliferation index was low (<10% within tumor islands); this estimate includes admixed background lymphocytes and therefore likely overestimates the tumor-intrinsic rate. The tumor exhibited a nested pattern with occasional vague tubular epithelial arrangements (H&E, 20x) (Figure 1B and Figure S2). The nuclei appeared round to slightly elongated with inconspicuous nucleoli. Only mild anisonucleosis was present, without significant nuclear pleomorphism, anaplasia, brisk mitotic activity, or necrosis (H&E, 40x) (Figure 1B). Immunohistochemical staining demonstrated diffuse positivity for CD56 and focal strong positivity for cytokeratin cocktail and S100 (Figure S2). *In situ* hybridization showed strong positive staining for GLI1, a transcription factor in the Sonic hedgehog (SHH) pathway (Figure S2).[18] Based on the morphology and immunoprofile, a diagnosis of GLI1-altered neoplasm was established. Chromosomal microarray analysis detected no significant genomic aberrations, including those on chromosome 12 near the *GLI1* gene.

A comprehensive metastatic workup, including positron emission tomography (PET), showed no evidence of systemic disease. A surveillance MRI 3 months after the biopsy showed a modest increase in tumor size (1.8 x 0.8 x 1.9 cm), with similar extension into the left aspect of the spinal canal (Figure S1). On subsequent serial imaging the mass then stabilized: it was unchanged on 9 January 2025 and again on the most recent study on 7 April 2025, approximately 7 months after biopsy, measuring approximately 2 cm at the left L2-L3 neural foramen with no new enhancing lesions (Figure 1A). The patient has remained clinically asymptomatic, with a normal neurologic examination and no deficits, and has received no antitumor treatment to date. Given the low-grade

histology, the sustained imaging stability, and the morbidity associated with chemotherapy, radiation, and surgical resection, management has consisted of active surveillance with serial imaging.

The patient was enrolled in the MiOncoseq clinical sequencing program for further molecular characterization.[19] Targeted panel sequencing confirmed a stable, diploid genome with no gross copy number changes or mutations in key tumor suppressor or oncogenes (Figure S3). RNA sequencing revealed no pathogenic gene fusion. Germline analysis, performed on the patient-derived tumor (peripheral blood being donor-derived), identified no pathogenic variant in cancer-predisposition genes. Notably, *GLI1* mRNA expression was markedly elevated, ranking in the 100th percentile of the institutional MiOncoSeq RNA-seq cohort, which served as the expression comparator because patient-matched negative-margin tissue was not available (the lesion involved the L2 nerve root and was biopsied rather than resected to preserve neurologic function) (Figure 1B). *PTCH1*, a canonical and relatively context-independent direct transcriptional target and negative-feedback gene of the GLI1/Sonic hedgehog pathway, was likewise expressed at the highest level in the cohort (100th percentile), providing functional evidence that the overexpressed GLI1 is transcriptionally active.

Given the rarity of GLI1-altered neoplasms and the patient's history of gene therapy, a potential link between AAV therapy and tumorigenesis was suspected but not evident in targeted short-read sequencing. To explore this further, we analyzed tumor DNA using our newly established long-read sequencing clinical pipeline (Supplementary Methods), designed to detect variants that may have been missed by short-read sequencing.

DNA extracted from frozen tissue was subjected to long-read sequencing using the Oxford Nanopore PromethION platform, yielding a read N50 of 11.34 kb and an 80X coverage depth. Haplotype phasing placed *GALC* p.Arg220Ter and p.Tyr319Cys on separate haplotypes, the compound-heterozygous configuration carried by the patient but not by the healthy cord-blood donor, confirming that the tumor is *recipient-derived* rather than donor-derived (Figure S4).

Structural variant analysis identified a heterogeneous 1,038 bp insertion within the first intron of *GLI1* (Figure 1C and Figure S5). This fragment consists of a portion of the AAV2 inverted terminal repeats (ITR), and elements of the CAG cassette (the CMV enhancer, chicken Actb promoter, first exon, and splice donor) (Figure 1C and Figure S5). The insertion is flanked by two 115 bp target site duplications (TSDs) (Figure S5), consistent with capture of the vector fragment at a pre-existing double-strand break,[13,20] although the precise repair mechanism could not be resolved. The insertion was detected in 44% of reads, consistent with a clonal heterozygous integration in the diploid tumor genome. The integration was also independently confirmed by PCR and Sanger sequencing, which detected the insertion and the AAV-GLI1 junction in tumor but not non-tumor DNA and yielded a junction sequence concordant with the long-read assembly (Figure S6).

This insertion is predicted to produce aberrant splicing of chicken *Actb* exon 1 into *GLI1* exon 2, which results in chimeric transcripts that drive full-length *GLI1* expression. RiboErase RNA-seq data further support this finding, showing a high number of reads spanning the chicken *Actb* and human *GLI1* junctions (Figure S7). Additionally, allele-specific carbon-5 cytosine (5mC) methylation analysis revealed that hypomethylation across the *GLI1* gene body only occurs on the allele carrying the insertion, but not the wild-type allele (Figure S8). These observations further establish the causal relationship between viral integration and *GLI1* overexpression.

To determine the fate of the remaining AAV vector sequences, we performed *de novo* genome assembly followed by BLAST searches for the *GALC* transgene, the polyA signal, and the AAV2 ITR from the assembled contigs. No significant hits were detected, suggesting that the original vector was either defective, or the remaining episomal fragments were lost during the size selection step of library preparation. Furthermore, RNA-seq analysis showed the high variant allele fractions for *GALC* variants (Figure S4), indicating a lack of functional transgene expression. The case was immediately reported to the treating physicians with a 10-day turnaround time, and regulatory authorities, including the U.S. Food and Drug Administration (FDA), were promptly notified of this unprecedented adverse event.

Supplementary Discussion

Adeno-associated virus vectors are generally regarded as safe for gene therapy. At the time of this study's initiation, no case of AAV-driven tumorigenesis had been definitively confirmed in humans.[12] Their relatively low immunogenicity and inability to replicate without a helper virus make them an ideal choice for delivering therapeutic genes in conditions such as Krabbe disease. However, the detection of wild-type AAV sequences in hepatocellular carcinoma patients has raised concerns regarding a potential oncogenic role.[21] While insertional mutagenesis has been observed in certain mouse models, human studies have not consistently indicated that AAV contributes to cancer development.[12]

Interestingly, wild-type AAV DNA was detected more frequently in non-tumor liver than in HCC tissue (18% vs 8% of tissues) and generally at low copy number, indicating that detection of AAV sequences alone does not establish causation.[22] Beyond mere detection, vector design also matters:

high-dose vectors containing the ubiquitous chicken β-actin (CBA) promoter were associated with a markedly increased incidence of hepatocellular carcinoma driven by integrations at the Rian locus, whereas vectors incorporating alternative liver-directed regulatory cassettes such as hAAT retained Rian integrations but did not produce hepatocellular carcinoma in the studied cohorts.[23] These findings implicate strong enhancer/promoter elements as important modifiers of insertional oncogenesis and are relevant to both the present case and the recently reported AAV-associated CNS malignancy, which involved a ubiquitous CAG expression cassette.

A notable case involved a hemophilia patient who developed HCC following AAV-based gene therapy, initially raising safety concerns; however, subsequent analysis confirmed that the vector was not the cause.[24] In addition, a case of a low-grade epithelial neoplasm has been reported in a patient with spinal muscular atrophy (SMA) 14 months after treatment with onasemnogene abeparvovec, which uses AAV9 and the closely related, self-complementary CB promoter variant (a CMV enhancer and chicken β-actin promoter carrying an SV40 intron rather than the chicken β-actin splice donor of the CAG cassette). In this tumor, there was detection of vector nucleic acid signals in the tumor cells; however, analysis for insertion site integration was inconclusive and unable to determine whether the vector signals in the tumor cells were integrated or episomal. Interestingly, like our patient, this tumor also had relative overexpression of *GLI1* and *PTCH1*.[25] There is also another case report of a pilocytic astrocytoma developing in a patient with SMA following the same gene therapy, but the analysis did not show conclusive evidence of clonal integration.[26] In May 2026, Ahrens-Nicklas and colleagues reported the first definitively confirmed case of AAV integration-driven oncogenesis: a neuroepithelial tumor in a 5-year-old boy with mucopolysaccharidosis type I, identified four years after intracisternal magna delivery of

an AAV9-*IDUA* vector (RGX-111), with clonal integration of rearranged AAV vector elements into the *PLAG1* oncogene.[15]

Our case complements these observations. The tumor belongs to the rare category of GLI1-altered neoplasms,[27] which are mesenchymal tumors characterized by recurrent gene fusions involving GLI1, a zinc-finger transcription factor that serves as the terminal nuclear effector of Hedgehog signaling. GLI1 expression is most prominent during embryonic development and is generally low in postnatal tissues; consequently, aberrant activation through amplification, gene fusion, or enhancer hijacking can be oncogenic.[28] These tumors typically display epithelioid or spindle cell morphology with nested growth patterns. They express S100 but lack SOX10 expression, distinguishing them from neural crest-derived neoplasms. While these tumors may share features with pericytomas or myoepithelial tumors, their distinct molecular profile and clinical course warrant classification as a separate pathological entity.[27] Notably, GLI1-altered neoplasms frequently involve human β-actin (*ACTB*) as a fusion partner.[27] In our case, ironically, the *GLI1* fusion partner was derived from chicken *Actb* introduced to the cell through the AAV vector.

We would like to acknowledge two limitations in our molecular characterization of the neoplasm. First, the precise cell of origin cannot be resolved from bulk tumor tissue obtained 26 months after gene therapy. The clonal heterozygous integration nonetheless places the event within the neoplastic clone, and haplotype phasing confirms recipient rather than donor origin. The location at the left L2 nerve root and the GLI1-altered mesenchymal phenotype together point to a recipient-derived local paraspinal or nerve-root-associated mesenchymal or progenitor cell, though finer lineage attribution would require single-cell, spatial, or sorted-cell analyses beyond the scope of this Correspondence. Second, a broader GLI1 target-gene signature was not pursued: no

comparable Sonic hedgehog-driven mesenchymal neoplasm exists in our clinical sequencing program as a reference, and Hedgehog/GLI target programs are highly cell-type specific,[28] so such a comparison would likely report lineage rather than pathway activity.

Our case and the Ahrens-Nicklas case[15] share a unifying mechanism but diverge in integration architecture and route of delivery (intravenous vs CNS-directed). Both vectors used a CAG promoter (a CMV enhancer plus a chicken β-actin promoter) with a chicken β-actin splice donor, and in both tumors the splice donor mediated aberrant chimeric transcription of a developmental transcription factor (*GLI1* and *PLAG1* respectively). Their integrant was not a full-length vector but a truncated, heavily rearranged structure comprising an inverted duplication of the CAG promoter-enhancer and CBA splice donor, fragments of the *IDUA* transgene, a nonvector chromosome-10 fragment of HEK293 producer-cell origin, and a duplicated *PLAG1* intron-4 segment; ours comprised a 1,038-bp subgenomic fragment lacking the *GALC* transgene and polyadenylation signal. This subgenomic fragment in our patient resembles the "snapback genomes" and promoter-only vectors that recent work has identified as a particularly concerning class of AAV manufacturing byproducts; such elements can activate proto-oncogenes through readthrough transcription or enhancer effects in mouse models, especially with strong, ubiquitous promoters such as the CAG cassette.[29] Because the clinical vector lot was unavailable for sequencing, we cannot determine whether this transgene-less species was a manufacturing byproduct or arose from in vivo processing after transduction, and we frame it as a *hypothesis*.

The convergence of these two cases highlights the risk posed when a vector splice donor encounters a strong host splice acceptor in an oncogene exon**.** This concern is amplified by the high frequency of AAV integration into intronic sites at DNA breaks, with one in vitro study

reporting up to 47% of AAV integrations occurring at Cas9-induced double-strand breaks.[30] The splicing cassette is commonly included in AAV vector design to enhance transgene expression,[31] but the splice donor it contains can splice into the next exon of an oncogene when integrated nearby. Without the splice donor, aberrant transcription could still extend into intronic regions, but translation would likely encounter a premature stop codon before reaching the next exon, reducing the risk of tumorigenesis. Future research should explore methods to enhance transgene expression without including a splicing cassette in the vector.

We are cautious in classifying this tumor as a conventional secondary malignancy of transplantation: post-HSCT secondary malignancies are predominantly hematologic, and the increased risk of solid and central nervous system tumors is associated chiefly with total-body or cranial irradiation, which this patient did not receive.[32] Instead, the two genotoxic, alkylator-based conditioning regimens are relevant in two complementary ways. First, as a source of DNA damage, including double-strand breaks, that may have facilitated AAV integration; given in vitro evidence that AAV exploits pre-existing breaks as integration sites,[13,30] the short interval between conditioning and vector administration (29 days after the second UCBT) may have increased this risk. Second, the accompanying profound immunosuppression, with poor immune reconstitution requiring DLI and subsequent GVHD treated with corticosteroids and ruxolitinib, may have impaired tumor immunosurveillance and permitted outgrowth of the transformed clone, a consideration also raised in the MPS I case.

Given these host factors, the absolute risk observed here should not be extrapolated to standard immunocompetent rAAV recipients, and both confirmed human cases to date arose after hematopoietic transplantation, so direct human evidence is currently limited to this context. The

integration mechanism itself is not inherently host-restricted, however, and whether a comparable risk applies to immunocompetent recipients remains unknown, warranting continued genomic surveillance across rAAV gene therapy programs.

Last but not least, our investigation highlights the advantages of long-read sequencing in detecting insertional mutagenesis. Traditional paired-end short-read methods, limited to approximately 350-bp fragment sizes, often miss longer insertions.[33] While attempts have been made to detect longer insertions from short-read sequencing, these approaches typically require prior knowledge of target sequences to identify viral integrations or retrotransposon insertions. In contrast, nanopore-based long-read sequencing enables comprehensive, unbiased detection of these events, as demonstrated by our analysis.

While our findings do not negate the overall safety profile of AAV vectors, they underscore the critical importance of careful vector design and long-term monitoring of gene therapy recipients. This case should inform future AAV vector development, ultimately leading to even safer gene therapy practices.

Supplementary Methods

Regulatory Oversight

All specimens analyzed for this report — fresh-frozen tumor tissue, matched peripheral blood, and buccal DNA — were obtained from a patient enrolled in the University of Michigan MiOncoSeq and PEDS_MiOncoSeq precision oncology programs after written informed consent under IRB-approved protocols (HUM00046018, HUM00067928, and HUM00056496). Assays were performed in our CLIA-certified clinical molecular diagnostics laboratory under validated standard operating procedures for clinical genomic testing. All molecular data were generated through these programs, and clinical information was retrieved from the patient's routine medical record as part of standard care; no procedures or data collection were undertaken specifically for this report beyond those covered by the above protocols.

Integrative clinical sequencing by the Illumina platform

Tumor genomic DNA and total RNA were co-extracted from fresh-frozen tissue using the AllPrep DNA/RNA/miRNA Kit (Qiagen), and matched germline DNA was isolated from peripheral blood or a buccal swab with the DNeasy Blood & Tissue Kit (Qiagen). The patient described here had previously been profiled on the Illumina short-read platform through the MiOncoSeq workflow, comprising targeted exon-capture sequencing and capture-based RNA sequencing, as detailed previously.[34–36]

Long-read sequencing protocol

The tumor genomic DNA aliquot reserved for long-read sequencing was first cleaned up with AMPure XP beads in 0.75 M NaCl / 20% PEG-8000 to deplete fragments shorter than approximately 2 kb. Fragment-length distribution was then profiled by pulsed-field gel electrophoresis on a Pippin Pulse instrument (Sage Science); when residual short fragments remained, an additional high-pass size selection was performed on the BluePippin platform (Sage Science) to retain molecules longer than 5 kb.

Nanopore libraries were constructed from 1 µg of high–molecular–weight DNA using the Ligation Sequencing Kit V14 (SQK-LSK114; Oxford Nanopore Technologies) in combination with the NEBNext Companion Module (E7180S; New England Biolabs), following the manufacturers' protocols and encompassing end repair, dA-tailing, and sequencing-adaptor ligation. The final library was purified with AMPure XP beads, quantified on a Qubit 3.0 Fluorometer (Thermo Fisher Scientific), and loaded onto an A100 R10.4.1 flow cell on a PromethION P24 instrument (Oxford Nanopore Technologies). Sequencing of the tumor specimen achieved a read N50 of 11.34 kb at approximately 80× depth.

RiboErase RNA sequencing

To capture splicing events with greater sensitivity than poly-A capture and capture-sequencing allows, tumor RNA was also processed with the KAPA RNA HyperPrep Kit with RiboErase (Roche), following the manufacturer's instructions. In brief, total RNA was first depleted of ribosomal RNA by RiboErase to enrich mRNA and non-coding species, after which fragmented RNA was reverse-transcribed and converted to double-stranded cDNA, followed by end repair, A-

tailing, adaptor ligation, and PCR enrichment. Final library size distributions and concentrations were verified on an Agilent Bioanalyzer prior to Illumina sequencing.

Short-read and long-read sequencing analyses

Illumina short-read data were generated on *NovaSeq* 6000 instruments, yielding 100–150 bp paired-end reads from libraries with approximately 350 bp average insert size. Per-base quality of raw reads was inspected with *FastQC*.[37] Reads were mapped to GRCh37/hg19 using *NovoAlign* (v4.02.02)[38] under default parameters tuned for clinical sequencing; alignments were sorted and de-duplicated with *NovoSort*,[38] followed by base-quality recalibration and local indel realignment per *GATK* Best Practices.[39]

For point-mutation and small-indel detection, *FreeBayes* (v1.3.6)[40] was used in parallel with *Pindel* (v0.2.5 b8)[41] which captured larger indels and intermediate-size structural events. Copy-number alterations together with joint tumor purity and ploidy were derived using our in-house *cnatools* pipeline.[34–36] RNA-seq reads were aligned by *STAR* (v2.7.10a)[42] in two-pass mode, and fusion transcripts were nominated independently by *Arriba* (v2.4.0)[43] and *STAR-Fusion* (v1.15.0).[44]

Long-read sequencing was performed on PromethION flow cells (R10.4.1 chemistry) with real-time basecalling by *Dorado*[45] in high-accuracy mode (model dna_r10.4.1_e8.2_400bps_hac@v5.0.0). Run-level metrics — read-length distribution, per-read qualities, and throughput — were summarized with *ToulligQC* (v2.7.1).[46]

Long-reads were aligned to GRCh38 using *minimap2* (v2.28)[47] with long-read presets (-ax map-ont). To resolve the AAV integration locus (Figure 1C in the Main Text), reads spanning the

junction region were realigned against a custom hybrid reference in which the inserted vector sequence was concatenated to GRCh38 at the integration coordinate. Variant calling employed chemistry-matched models (r1041_e82_400bps_hac_v420): *Clair3* (v1.0.11)[48] for germline calls and *ClairS* (v0.4.1)[49] for somatic calls. Normalization and filtering of VCFs were performed with *bcftools* (v1.20).[50] Read-based phasing was performed on the matched-normal BAM with *WhatsHap* (v2.7)[51] restricted to high-confidence SNVs.

Long-read structural variants were detected with *Severus* (v1.5)[52] and filtered against (i) a cohort-derived blacklist of recurrent technical breakpoints (collapsed within a 100 bp window) and (ii) published noisy-region annotations.[53] Cytosine modifications (5mC and 5hmC) were emitted during basecalling using the 5mCG_5hmC model, and allele-resolved and differential-methylation analyses were carried out with *modkit* (v0.4.4).[54]

Supplementary Figures

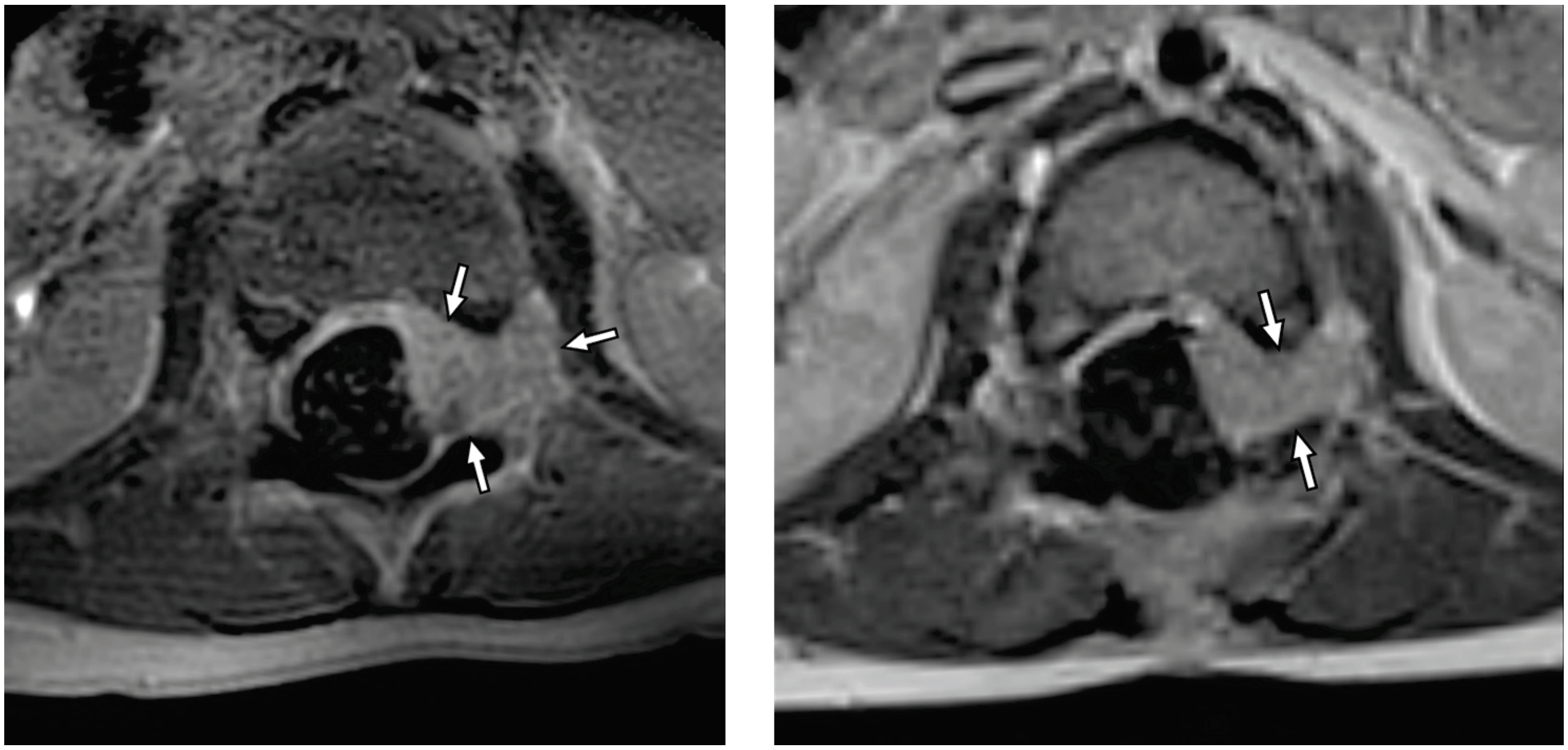

Figure S1: Additional MRI results of the spinal mass.

Left: Axial post-contrast enhanced MRI demonstrates homogeneous enhancement within the mass (arrows). **Right:** Follow-up MRI, obtained three months later, shows a relatively stable appearance of the lesion (arrows). Post-surgical changes are observed in the posterior spinous region following an interval biopsy.

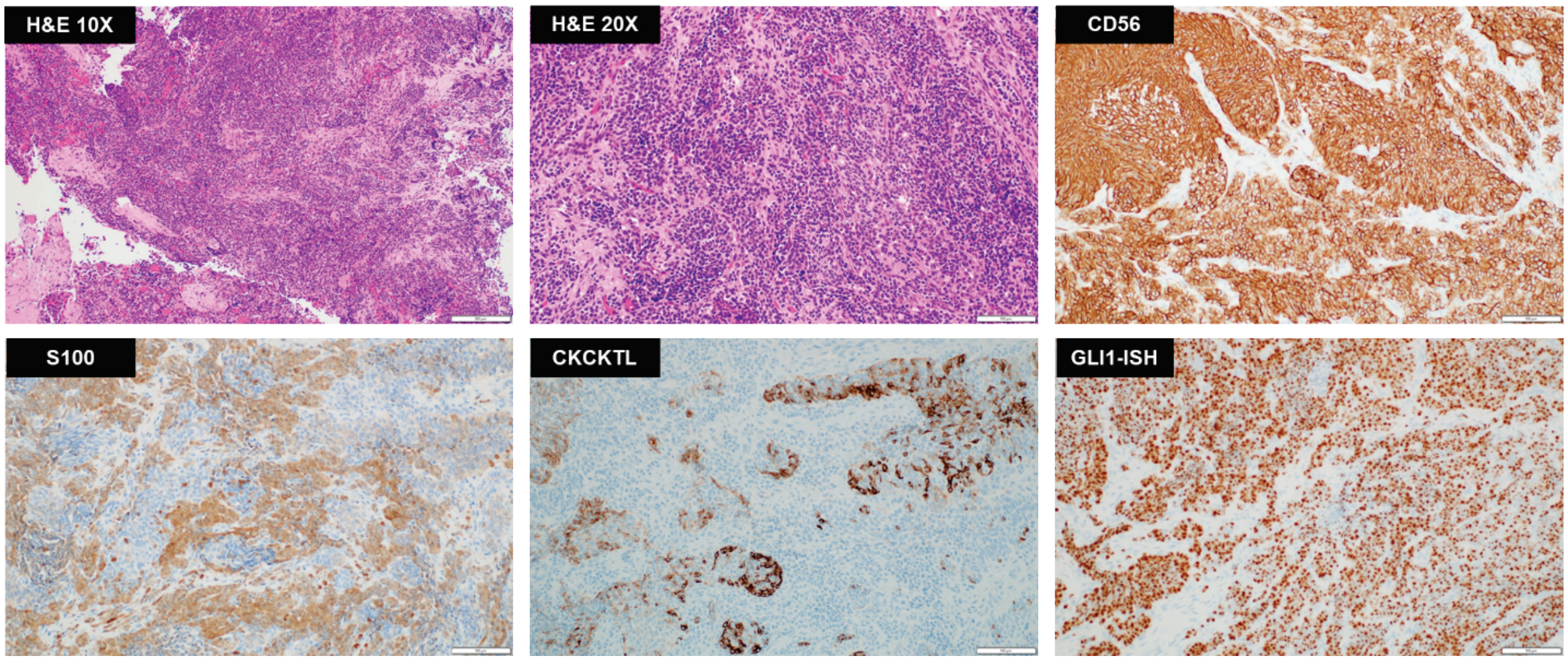

Figure S2: Tumor Histopathology and Immunohistochemistry.

Histological examination reveals a relatively monotonous small round blue cell neoplasm with focal epithelioid morphology infiltrating ganglion and soft tissue (H&E, 10×). The tumor exhibits a nested growth pattern with occasional vague tubular epithelial arrangements (H&E, 20×). Immunohistochemical analysis demonstrates diffuse CD56 positivity and focal strong positivity for cytokeratin cocktail (CKCKTL) and S100 protein. In situ hybridization confirms strong positive staining for GLI1, consistent with GLI1-altered neoplasm classification.

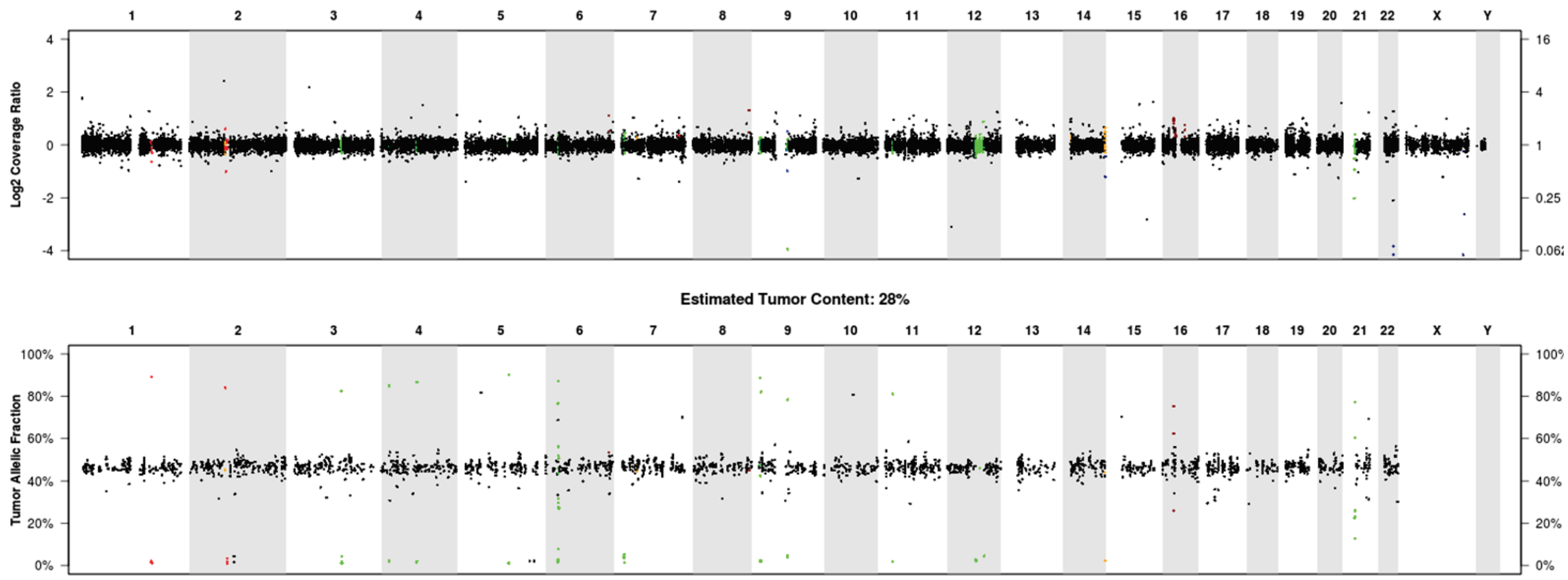

Figure S3: Short-read copy number analysis.

Copy number analysis was performed as previously described.[34-36] **Top:** Log2 coverage ratio of tumor versus pooled normal. **Bottom:** B-allelic frequency (BAF) of heterozygous single nucleotide polymorphisms (SNPs).

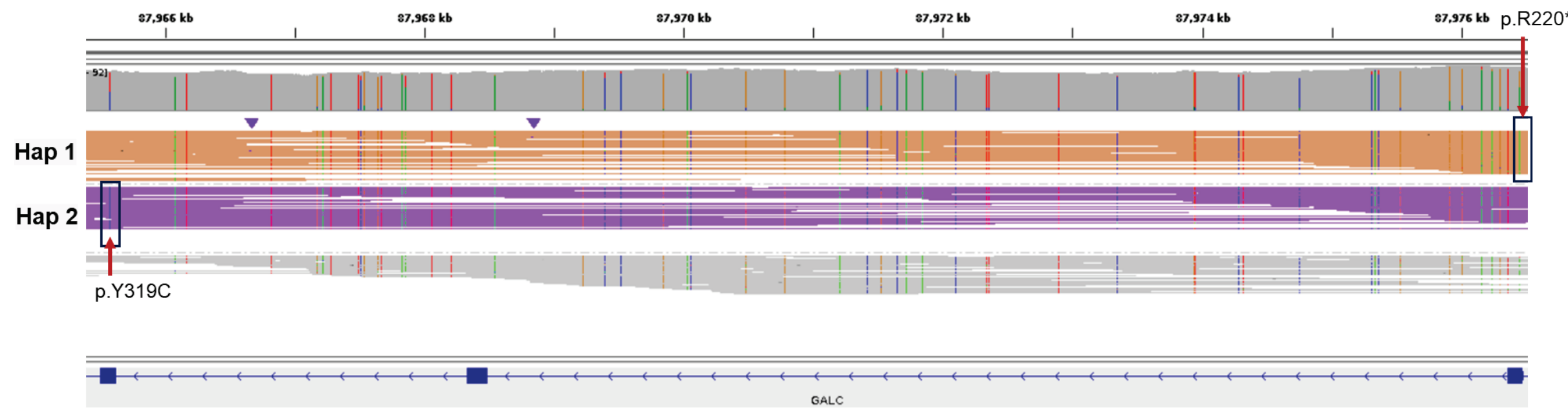

GALC Mutations	VAF Nanopore	VAF RNA-seq
p.R220*	58%	11%
p.Y319C	48%	72%

Figure S4: Haplotype phasing for *GALC* variants.

Top: Haplotype phasing of long-read sequencing data shows that the pathogenic *GALC* variants (p.R220* and p.Y319C) reside on different haplotypes. **Bottom:** Variant allelic fraction (VAF) of *GALC* variants in nanopore DNA sequencing and Illumina RNA sequencing. The high VAF of p.Y319C in RNA-Seq suggests that the transgene *GALC*, with the corrected sequence, is unlikely to be expressed. In contrast, the low VAF of p.R220* in RNA-Seq is likely due to nonsense-mediated decay.

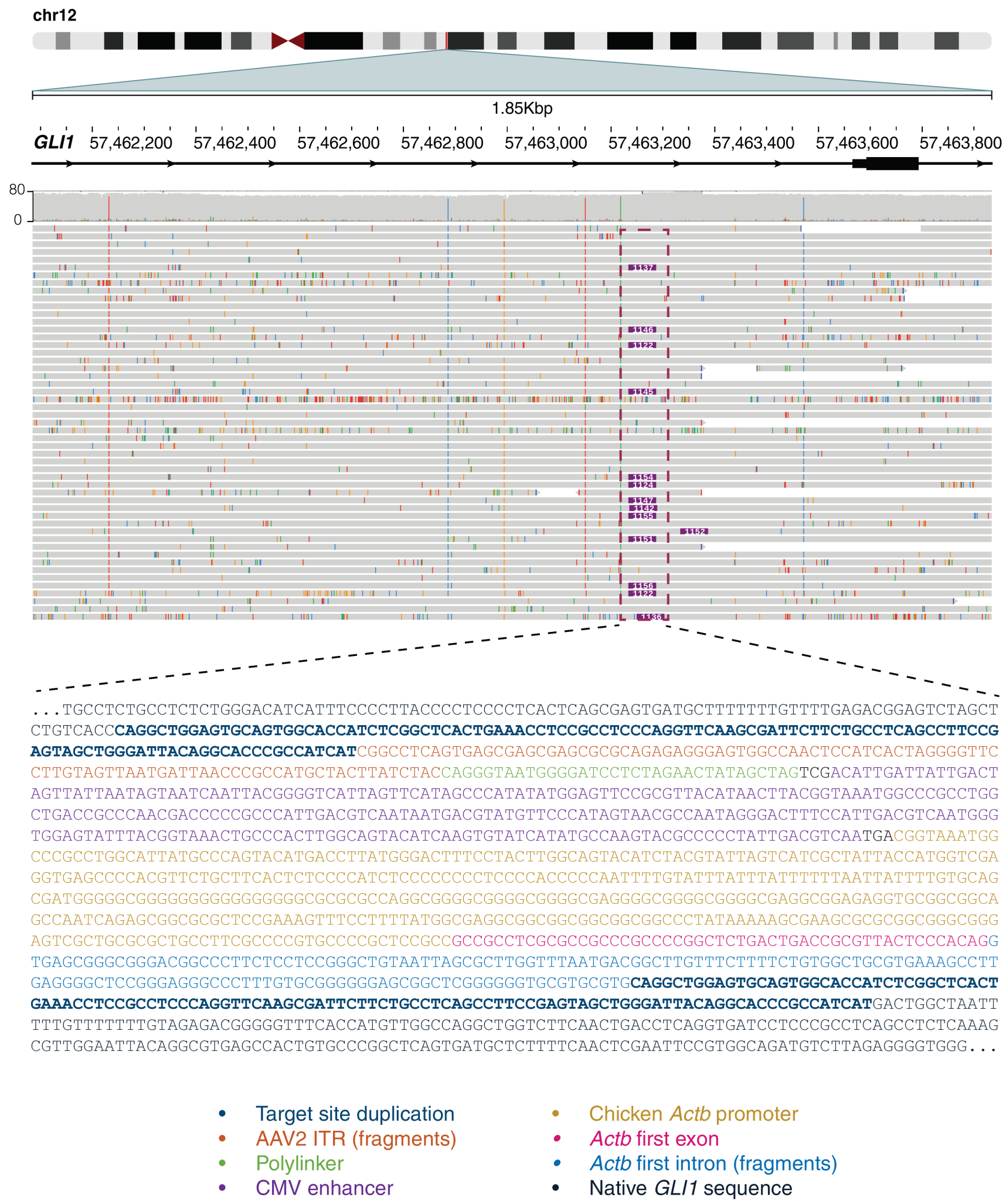

Figure S5: Genomic characterization of AAV vector integration.

Top: JBrowse visualization of the AAV insertion locus within *GLI1* intron 1, showing long-read alignment evidence and structural genomic context. The integration site demonstrates clonal insertion of vector sequences with characteristic junction patterns. **Bottom:** Detailed genomic sequence analysis reveals key functional elements of the integrated AAV fragment, including inverted terminal repeat (ITR) sequences, cytomegalovirus enhancer elements, chicken β-actin (*Actb*) promoter, *Actb* first exon, and splice donor sequences that drive aberrant *GLI1* activation.

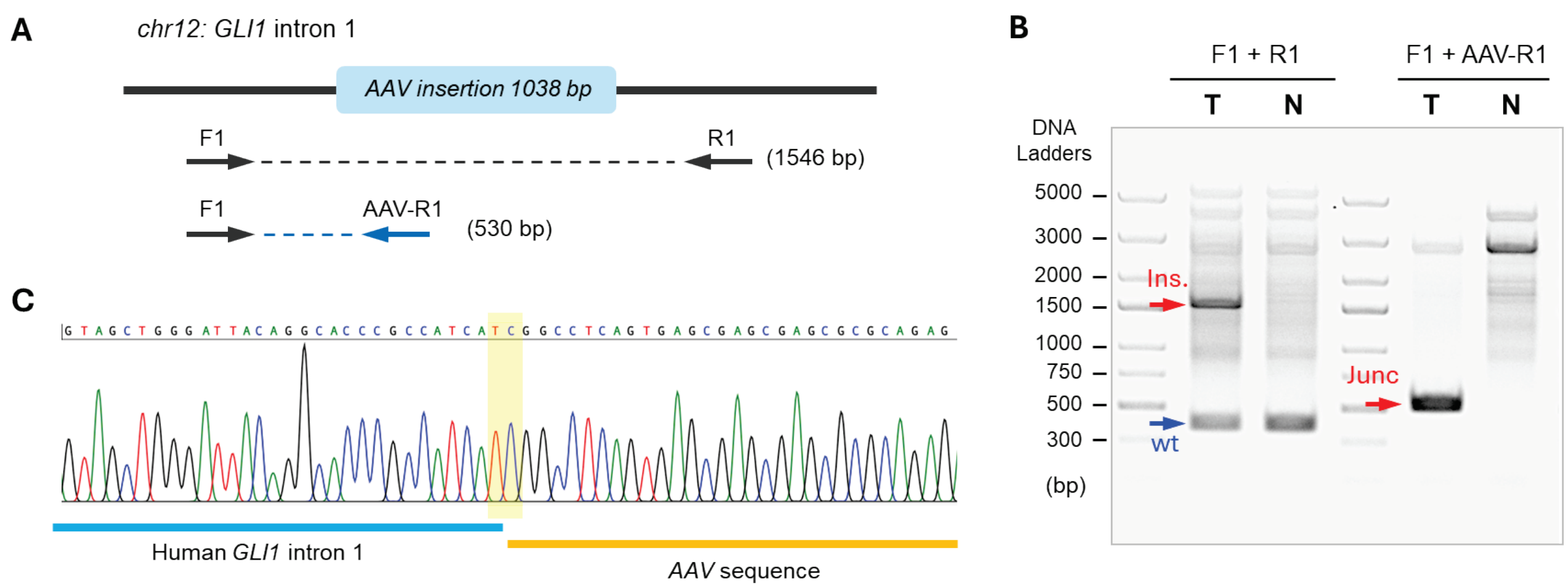

Figure S6: Orthogonal validation of rAAV insertion by PCR

PCR validation of AAV integration at the GLI1 locus, using genomic DNA from the patient's tumor (T) and matched non-tumor peripheral blood (N). **(A)** Two primer pairs: (1) F1 and R1, both in human genomic sequence flanking the insertion site, amplifying a 1,546-bp product from the insertion-bearing allele and a 393-bp product from the wild-type allele (wt); the 1,153-bp difference corresponds to the 1,038-bp integrant plus the flanking 115-bp target-site duplication; and (2) F1 with an AAV-specific reverse primer (AAV-R1), amplifying a 530-bp product only across an AAV-GLI1 junction. **(B)** Agarose gel electrophoresis. The tumor showed both the 1,546-bp insertion and 393-bp wild-type bands, consistent with clonal heterozygous integration, together with the 530-bp junction product; normal blood showed only the wild-type band and no junction product, confirming tumor-specific integration. **(C)** Sanger sequencing of the 530-bp junction product confirms the boundary between human GLI1 intron 1 and AAV vector sequence.

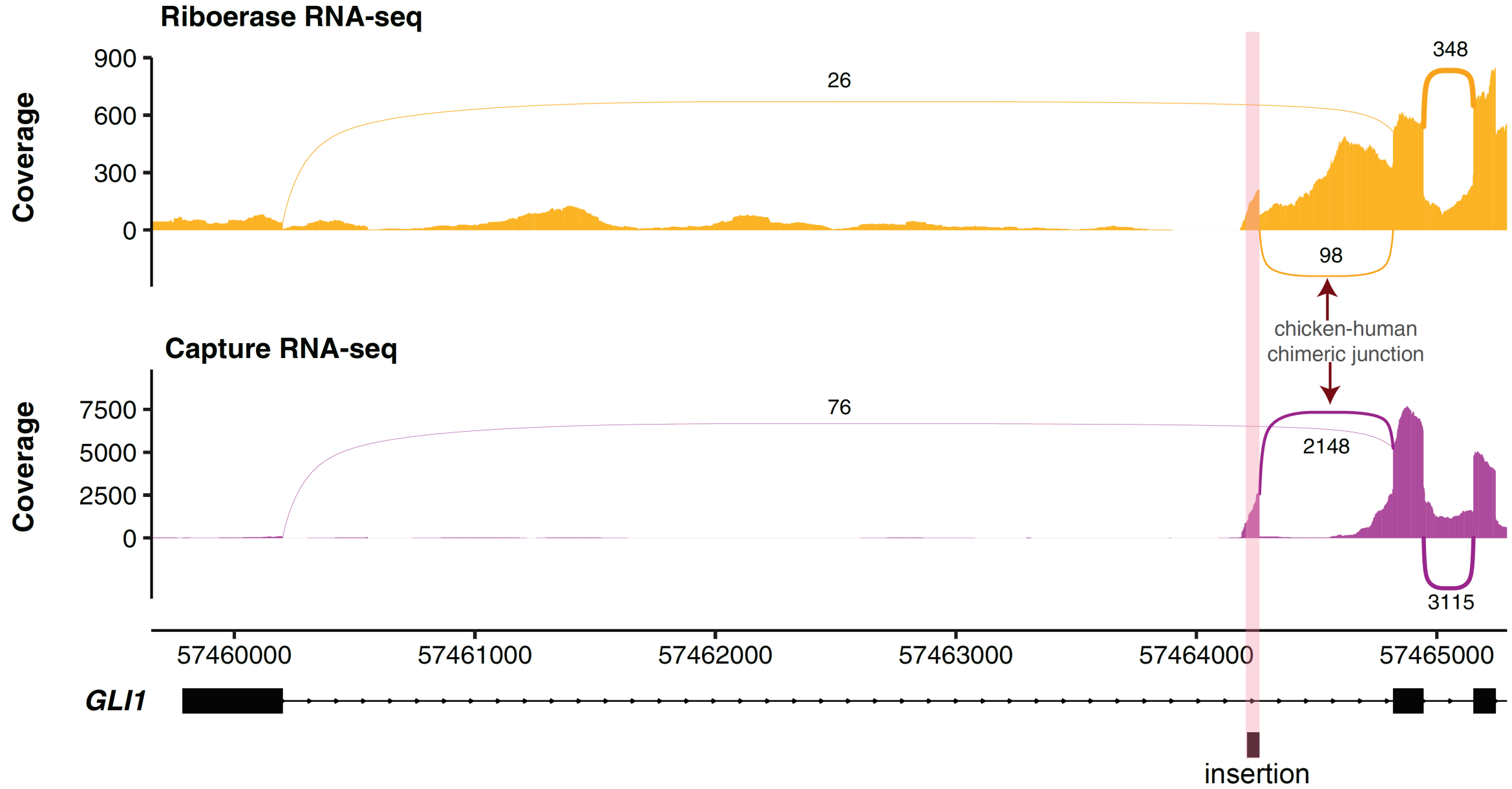

Figure S7: RNA sequencing analysis confirms aberrant GLI1 splicing

Sashimi plots illustrate *GLI1* splicing patterns detected by RiboErase RNA-seq (top) and standard capture-based RNA-seq (bottom) methodologies. RNA-seq libraries were aligned to a customized reference genome incorporating both hg38 and the AAV insertion sequence. Coverage mapping reveals expression of the chicken *Actb* first exon within the viral insertion (highlighted region). Read abundance supports the presence of chimeric *Actb-GLI1* splice junctions, confirming the mechanistic basis for *GLI1* overexpression. Interestingly, only low-level endogenous splicing between *GLI1* exons 1 and 2 is observed; this faint signal may derive from admixed surrounding non-tumorous tissue and is consistent with little or no endogenous *GLI1* expression in the tumor's cell of origin, in contrast to the abundant vector-driven chimeric transcript.

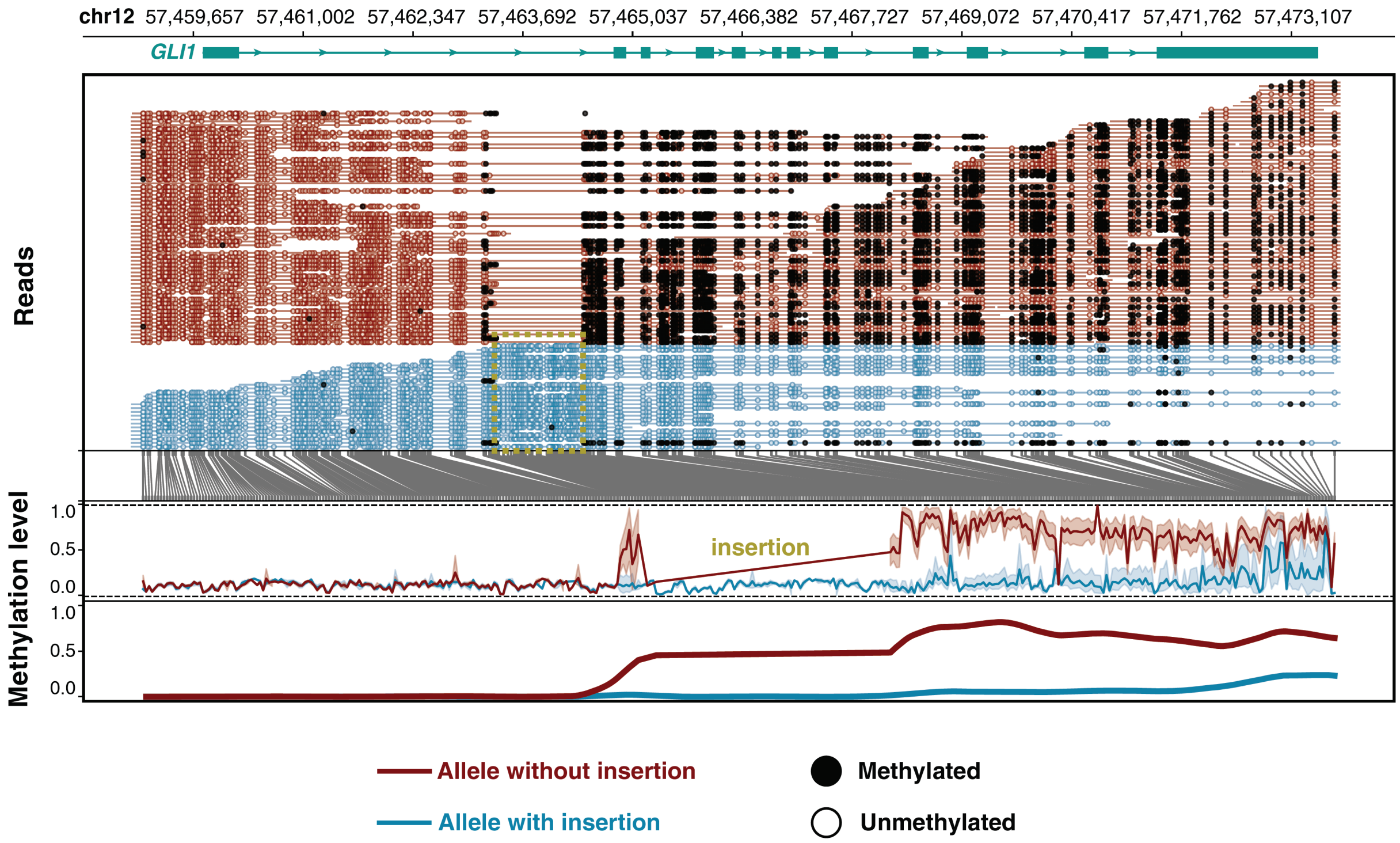

Figure S8: Allele-specific methylation analysis of the AAV integration site.

Nanopore sequencing with base modification detection was performed using reads aligned to a customized reference genome hg38 incorporating the AAV insertion. **Top to bottom:** aligned read view (black circles: methylated, white circles: non-methylated), raw methylation signal of individual CpG sites, smoothed methylation signal of individual CpG sites. Methylation analysis demonstrates allele-specific hypomethylation restricted to the chromosome harboring the AAV integration, while the non-integrated allele maintains normal gene-body methylation patterns. This epigenetic asymmetry provides additional evidence supporting a causal relationship between viral integration and *GLI1* transcriptional activation.

Supplementary References

1. Bradbury AM, Bongarzone ER, Sands MS. Krabbe disease: New hope for an old disease. *Neurosci Lett*. 2021;752:135841. doi:10.1016/j.neulet.2021.135841
2. Orsini JJ, Escolar ML, Wasserstein MP, et al. Krabbe Disease. 2000 Jun 19 [Updated 2018 Oct 11]. In: Adam MP, Feldman J, Mirzaa GM, et al., editors. GeneReviews® [Internet]. Seattle (WA): University of Washington, Seattle; 1993-2025. Available from: https://www.ncbi.nlm.nih.gov/books/NBK1238/
3. Wright MD, Poe MD, DeRenzo A, Haldal S, Escolar ML. Developmental outcomes of cord blood transplantation for Krabbe disease: A 15-year study. *Neurology*. 2017;89(13):1365-1372. doi:10.1212/WNL.0000000000004418
4. Siddiqi ZA, Sanders DB, Massey JM. Peripheral neuropathy in Krabbe disease: effect of hematopoietic stem cell transplantation. *Neurology*. 2006;67(2):268-272. doi:10.1212/01.wnl.0000230156.01228.33
5. Escolar ML, Poe MD, Provenzale JM, et al. Transplantation of umbilical-cord blood in babies with infantile Krabbe's disease. *N Engl J Med*. 2005;352(20):2069-2081. doi:10.1056/NEJMoa042604
6. Allewelt H, Taskindoust M, Troy J, et al. Long-Term Functional Outcomes after Hematopoietic Stem Cell Transplant for Early Infantile Krabbe Disease. *Biol Blood Marrow Transplant*. 2018;24(11):2233-2238. doi:10.1016/j.bbmt.2018.06.020
7. Hordeaux J, Jeffrey BA, Jian J, et al. Efficacy and Safety of a Krabbe Disease Gene Therapy. *Hum Gene Ther*. 2022;33(9-10):499-517. doi:10.1089/hum.2021.245
8. Liu D, Zhu M, Zhang Y, Diao Y. Crossing the blood-brain barrier with AAV vectors. *Metab Brain Dis*. 2021;36(1):45-52. doi:10.1007/s11011-020-00630-2
9. Marshall MS, Issa Y, Jakubauskas B, et al. Long-Term Improvement of Neurological Signs and Metabolic Dysfunction in a Mouse Model of Krabbe's Disease after Global Gene Therapy. *Mol Ther*. 2018;26(3):874-889. doi:10.1016/j.ymthe.2018.01.009
10. Bradbury AM, Bagel J, Swain G, et al. Combination HSCT and intravenous AAV-mediated gene therapy in a canine model proves pivotal for translation of Krabbe disease therapy. *Mol Ther*. 2024;32(1):44-58. doi:10.1016/j.ymthe.2023.11.014

11. Lek A, Wong B, Keeler A, Blackwood M, Ma K, Huang S, et al. Death after high-dose rAAV9 gene therapy in a patient with Duchenne's muscular dystrophy. *N Engl J Med*. 2023 Sept 28;389(13):1203–10.
12. Büning H, Schmidt M. Adeno-associated Vector Toxicity-To Be or Not to Be?. *Mol Ther*. 2015;23(11):1673-1675. doi:10.1038/mt.2015.182
13. Miller DG, Petek LM, Russell DW. Adeno-associated virus vectors integrate at chromosome breakage sites. *Nat Genet*. 2004;36(7):767-773. doi:10.1038/ng1380
14. Dalwadi DA, Torrens L, Abril-Fornaguera J, et al. Liver Injury Increases the Incidence of HCC following AAV Gene Therapy in Mice. *Mol Ther*. 2021;29(2):680-690. doi:10.1016/j.ymthe.2020.10.018
15. Ahrens-Nicklas RC, Kotch C, Roche AM, Everett JK, Reddy S, Santi M, et al. Neuroepithelial tumor with AAV integration after intracisternal magna vector delivery. *N Engl J Med*. 2026 May 13. doi:10.1056/NEJMoa2601608
16. U.S. Food and Drug Administration. Long term follow-up after administration of human gene therapy products. 2020. Available from: https://www.fda.gov/regulatory-information/search-fda-guidance-documents/long-term-follow-after-administration-human-gene-therapy-products
17. Heller G, Bradbury AM, Sands MS, Bongarzone ER. Preclinical studies in Krabbe disease: A model for the investigation of novel combination therapies for lysosomal storage diseases. *Mol Ther*. 2023 Jan 4;31(1):7–23.
18. Villavicencio EH, Walterhouse DO, Iannaccone PM. The sonic hedgehog-patched-gli pathway in human development and disease. *Am J Hum Genet*. 2000;67(5):1047-1054. doi:10.1016/S0002-9297(07)62934-6
19. Mody RJ, Wu YM, Lonigro RJ, et al. Integrative Clinical Sequencing in the Management of Refractory or Relapsed Cancer in Youth. *JAMA*. 2015;314(9):913-925. doi:10.1001/jama.2015.10080
20. Conrad DF, Bird C, Blackburne B, et al. Mutation spectrum revealed by breakpoint sequencing of human germline CNVs. *Nat Genet*. 2010;42(5):385-391. doi:10.1038/ng.564

21. Nault JC, Datta S, Imbeaud S, et al. Recurrent AAV2-related insertional mutagenesis in human hepatocellular carcinomas. *Nat Genet*. 2015;47(10):1187-1193. doi:10.1038/ng.3389
22. La Bella T, Imbeaud S, Peneau C, Mami I, Datta S, Bayard Q, et al. Adeno-associated virus in the liver: natural history and consequences in tumour development. *Gut*. 2020 Apr;69(4):737–47.
23. Chandler RJ, LaFave MC, Varshney GK, Trivedi NS, Carrillo-Carrasco N, Senac JS, et al. Vector design influences hepatic genotoxicity after adeno-associated virus gene therapy. *J Clin Invest*. 1 2015;125(2):870–80.
24. Kaiser, J. (2021, February 1). *Experimental gene therapy for hemophilia probably did not cause patient's liver tumor*. *Science*. Retrieved from https://www.science.org/content/article/experimental-gene-therapy-hemophilia-probably-did-not-cause-patient-s-liver-tumor
25. Retson L, Tiwari N, Vaughn J, et al. Epithelioid neoplasm of the spinal cord in a child with spinal muscular atrophy treated with onasemnogene abeparvovec. *Mol Ther*. 2023;31(10):2991-2998. doi:10.1016/j.ymthe.2023.08.013
26. Holzwarth D, Calaminus G, Friese J, Sejersen T, Büning H, John-Neek P, et al. Pilocytic astrocytoma in a child with spinal muscular atrophy treated with onasemnogene abeparvovec. *Mol Ther.* 2025 June 4;33(6):2842–50.
27. Antonescu CR, Agaram NP, Sung YS, Zhang L, Swanson D, Dickson BC. A Distinct Malignant Epithelioid Neoplasm With GLI1 Gene Rearrangements, Frequent S100 Protein Expression, and Metastatic Potential: Expanding the Spectrum of Pathologic Entities With ACTB/MALAT1/PTCH1-GLI1 Fusions. *Am J Surg Pathol*. 2018;42(4):553-560. doi:10.1097/PAS.0000000000001010
28. Ruiz i Altaba A, Sánchez P, Dahmane N. Gli and hedgehog in cancer: tumours, embryos and stem cells. *Nat Rev Cancer*. 2002 May;2(5):361–72.
29. Brimble MA, Winston SM, Davidoff AM. Stowaways in the cargo: Contaminating nucleic acids in rAAV preparations for gene therapy. *Mol Ther*. 2023;31(10):2826-2838. doi:10.1016/j.ymthe.2023.07.025

30. Hanlon KS, Kleinstiver BP, Garcia SP, et al. High levels of AAV vector integration into CRISPR-induced DNA breaks. *Nat Commun.* 2019;10(1):4439. Published 2019 Sep 30. doi:10.1038/s41467-019-12449-2
31. Wang D, Tai PWL, Gao G. Adeno-associated virus vector as a platform for gene therapy delivery. *Nat Rev Drug Discov*. 2019 May;18(5):358–78.
32. Rizzo JD, Curtis RE, Socié G, Sobocinski KA, Gilbert E, Landgren O, et al. Solid cancers after allogeneic hematopoietic cell transplantation. *Blood*. 2009 Jan 29;113(5):1175–83.
33. Lee YG, Lee JY, Kim J, Kim YJ. Insertion variants missing in the human reference genome are widespread among human populations. *BMC Biol*. 2020;18(1):167. Published 2020 Nov 13. doi:10.1186/s12915-020-00894-1
34. Robinson D, Van Allen EM, Wu Y-M, Schultz N, Lonigro RJ, Mosquera J-M, et al. Integrative clinical genomics of advanced prostate cancer. *Cell*. 2015 May 21;161(5):1215–28.
35. Robinson DR, Wu Y-M, Lonigro RJ, Vats P, Cobain E, Everett J, et al. Integrative clinical genomics of metastatic cancer. *Nature*. 2017 Aug 17;548(7667):297–303.
36. Cobain EF, Wu Y-M, Vats P, Chugh R, Worden F, Smith DC, et al. Assessment of clinical benefit of integrative genomic profiling in advanced solid tumors. *JAMA Oncol*. 2021 Apr 1;7(4):525–33.
37. Andrews S. FastQC: A quality control tool for high throughput sequence data [Internet]. Cambridge, UK: Babraham Bioinformatics; 2010 [cited 2025 Jul 26]. Available from: http://www.bioinformatics.babraham.ac.uk/projects/fastqc/
38. Novocraft Technologies. *NovoAlign v4.02.02 and NovoSort* [Internet]. Selangor, Malaysia: Novocraft Technologies Sdn Bhd; [cited 2025 Jul 26]. Available from: http://www.novocraft.com
39. Van der Auwera GA, Carneiro M, Hartl C, Poplin R, del Angel G, Levy-Moonshine A, Jordan T, Shakir K, Roazen D, Thibault J, Banks E, Garimella K, Altshuler D, Gabriel S, DePristo M. From FastQ Data to High-Confidence Variant Calls: The Genome Analysis Toolkit Best Practices Pipeline. *Curr Protoc Bioinformatics*. 2013;43:11.10.1-11.10.33.
40. Garrison E, Marth G. Haplotype-based variant detection from short-read sequencing [Internet]. arXiv [q-bio.GN]. 2012. Available from: http://arxiv.org/abs/1207.3907

41. Ye K, Schulz MH, Long Q, Apweiler R, Ning Z. Pindel: a pattern growth approach to detect break points of large deletions and medium sized insertions from paired-end short reads. *Bioinformatics*. 2009 Nov 1;25(21):2865–71.
42. Dobin A, Davis CA, Schlesinger F, Drenkow J, Zaleski C, Jha S, et al. STAR: ultrafast universal RNA-seq aligner. *Bioinformatics*. 2013 Jan 1;29(1):15–21.
43. Uhrig S, Ellermann J, Walther T, Burkhardt P, Fröhlich M, Hutter B, et al. Accurate and efficient detection of gene fusions from RNA sequencing data. *Genome Res*. 2021 Mar;31(3):448–60.
44. Haas BJ, Dobin A, Stransky N, Li B, Yang X, Tickle T, et al. STAR-fusion: Fast and accurate fusion transcript detection from RNA-Seq [Internet]. bioRxiv. bioRxiv; 2017. Available from: http://dx.doi.org/10.1101/120295
45. Oxford Nanopore Technologies. Dorado basecaller [Internet]. Oxford, UK: Oxford Nanopore Technologies Ltd; [cited 2025 Jul 26]. Available from: https://github.com/nanoporetech/dorado
46. GenomiqueENS (École normale supérieure, IBENS). ToulligQC: automated QC pipeline for Oxford Nanopore runs [Internet]. Paris (FR): GenomiqueENS; 2018 [cited 2025 Jul 26]. Available from: https://github.com/GenomiqueENS/toulligQC
47. Li H. Minimap2: pairwise alignment for nucleotide sequences. *Bioinformatics*. 2018 Sep 15;34(18):3094–100.
48. Zheng Z, Li S, Su J, Leung AW-S, Lam T-W, Luo R. Symphonizing pileup and full-alignment for deep learning-based long-read variant calling. *Nat Comput Sci*. 2022 Dec;2(12):797–803.
49. The University of Hong Kong, Bioinformatics Algorithms & Core Technology Lab. ClairS v0.1.5 [Internet]. Hong Kong: The University of Hong Kong; 2023 Aug 2 [cited 2025 Jul 26]. Available from: https://github.com/HKU-BAL/ClairS
50. Li H. A statistical framework for SNP calling, mutation discovery, association mapping and population genetical parameter estimation from sequencing data. *Bioinformatics*. 2011 Nov 1;27(21):2987–93.

51. Martin M, Patterson M, Garg S, O Fischer S, Pisanti N, Klau GW, et al. WhatsHap: fast and accurate read-based phasing [Internet]. bioRxiv. bioRxiv; 2016. Available from: http://dx.doi.org/10.1101/085050
52. Keskus A, Bryant A, Ahmad T, Yoo B, Aganezov S, Goretsky A, et al. Severus: accurate detection and characterization of somatic structural variation in tumor genomes using long reads. medRxiv [Internet]. 2024 Mar 26; Available from: http://dx.doi.org/10.1101/2024.03.22.24304756
53. Tobias Rausch et al.; Dellytools. DELLY v1.1.6 [Internet]. Heidelberg, Germany: dellytools; [cited 2025 Jul 26]. Available from: https://github.com/dellytools/delly
54. Oxford Nanopore Technologies. *Modkit v0.4.4* [Internet]. Cambridge, UK: Oxford Nanopore Technologies; 2024 Sep 7 [cited 2025 Jul 26]. Available from: https://github.com/nanoporetech/modkit
